# The link between chronic periodontitis and COPD: a common role for the neutrophil?

**DOI:** 10.1186/1741-7015-11-241

**Published:** 2013-11-13

**Authors:** Adam KH Usher, Robert A Stockley

**Affiliations:** 1 Lung Investigation Unit, Queen Elizabeth Hospital, Mindelsohn Way, Edgbaston, Birmingham B15 2WB, UK

**Keywords:** Chronic obstructive pulmonary disease, Emphysema, Neutrophil, Neutrophil extracellular trap, Oxidative stress, Periodontal diseases, Protease/proteinase

## Abstract

**Background:**

The possible relationship between chronic inflammatory diseases and their co-morbidities has become an increasing focus of research. Both chronic periodontitis and chronic obstructive pulmonary disease are neutrophilic, inflammatory conditions characterized by the loss of local connective tissue. Evidence suggests an association and perhaps a causal link between the two diseases. However, the nature of any relationship between them is unclear, but if pathophysiologically established may have wide-reaching implications for targeted treatments to improve outcomes and prognosis.

**Discussion:**

There have been a number of epidemiological studies undertaken demonstrating an independent association between chronic periodontitis and chronic obstructive pulmonary disease. However, many of them have significant limitations, and drawing firm conclusions regarding causality may be premature. Although the pathology of both these diseases is complex and involves many cell types, such as CD8 positive cells and macrophages, both conditions are predominantly characterized by neutrophilic inflammation. Increasingly, there is evidence that the two conditions are underpinned by similar pathophysiological processes, especially centered on the functions of the neutrophil. These include a disturbance in protease/anti-protease and redox state balance. The association demonstrated by epidemiological studies, as well as emerging similarities in pathogenesis at the level of the neutrophil, suggest a basis for testing the effects of treatment for one condition upon the severity of the other.

**Summary:**

Although the evidence of an independent association between chronic periodontitis and chronic obstructive pulmonary disease grows stronger, there remains a lack of definitive studies designed to establish causality and treatment effects. There is a need for future research to be focused on answering these questions.

## Background

Periodontitis is a common infectious disease of the mouth affecting the supporting structures of the teeth. Around half of adults are affected by significant periodontal pocket and attachment loss [1], with around 11% of adults having chronic periodontitis [2]. Although chronic periodontitis involves a complex interplay of cytokines and cell types, it is mainly considered a neutrophil-mediated disease [3]. Plaque build-up allows the growth of anaerobic bacteria [4], which via a series of mechanisms leads to the recruitment and activation of neutrophils. Excessive or unopposed exposure of the connective tissue to the neutrophils’ enzymes causes its destruction. Left untreated, chronic periodontitis causes loss of ligamentous support and alveolar bone, resulting in tooth loss [5].

Chronic obstructive pulmonary disease (COPD) affects around 200 million people worldwide and is a major cause of morbidity and mortality [6]. It is important to emphasize that COPD is a generic term that is defined by the presence of airflow obstruction. It consists of several pathological subtypes, such as emphysema, small airways disease and chronic bronchitis, that are distinct entities although often combined in a single patient. Throughout this review, COPD is used unless distinct pathological entities are known. COPD is a complex disease involving many types of immune responses that recruit many types of innate and adaptive immune cells, as well as the potential involvement of autoantibodies [7]. As with chronic periodontitis, it is recognized predominantly as a chronic neutrophilic inflammatory disorder and enzymes from the neutrophil granules are implicated in the pathogenesis of the disease [8].

**Figure 1:**
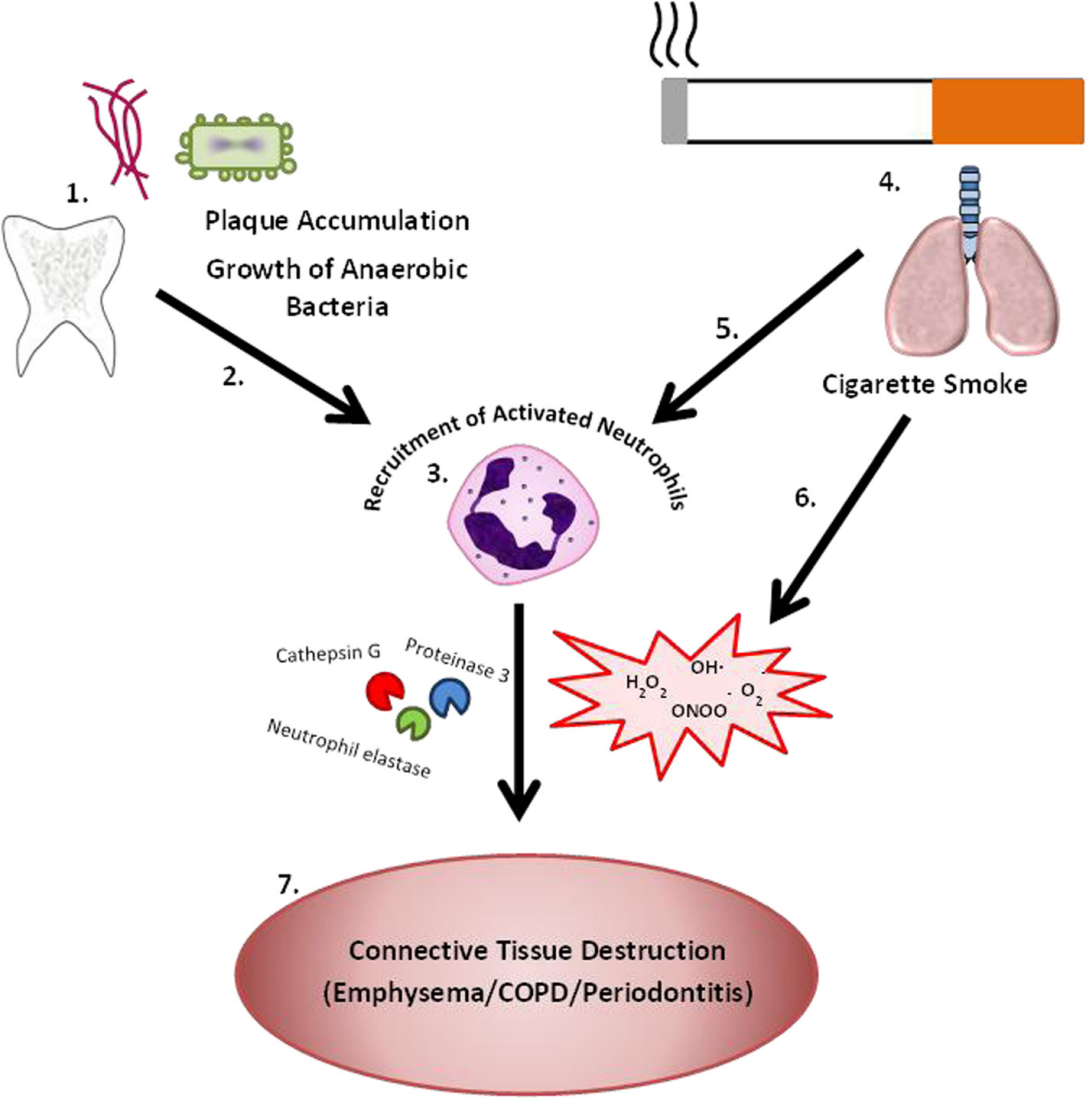
**Convergence of the principal triggers of inflammation for periodontitis and common obstructive pulmonary disease on a common pathophysiological process involving neutrophil activation.** In the dento-gingival cavity, plaque accumulation leads to the growth of bacteria such as *Porphyromonas gingivalis* and *Fusobacterium nucleatum* (1). The release of bacterial proteins and induction of cytokine expression (2) lead to the recruitment of activated neutrophils (3). Particulate matter from cigarette smoke (4) causes the local production of inflammatory cytokines, also leading to the local accumulation of activated neutrophils (5) and providing an oxidant stress to the local tissues (6). The products from inflammatory cells including chemoattractants, proteases and reactive oxygen species can amplify the inflammatory process whilst causing the connective tissue damage seen at both sites (7). The susceptibility to either pathology depends on a heightened downstream process, which may have a common abnormality that makes it more likely for both diseases to develop. COPD, common obstructive pulmonary disease.

Both chronic periodontitis and COPD have a common element of host susceptibility to environmental factors. For example, it is widely accepted that smoking is a risk factor for developing COPD, however the disease only affects a minority of smokers, implying intrinsic and genetic factors that may negate the effect of smoking [9]. Similarly, a complex interaction of environmental and genetic factors has been described in periodontitis [5]. It has been hypothesized that, despite some variations in the triggers and possibly susceptibility factors, the pathological mechanism in both diseases converges on activating and utilizing neutrophils. In both diseases, the released neutrophil molecules have the capacity to cause the pathological changes seen in connective tissue [10], as summarized below, leading to two identical processes in different tissues with a chronic destructive outcome (Figure 1).

There has been growing interest in the hypothesis that COPD forms part of a ‘chronic systemic inflammatory syndrome’ [11]. Patients with COPD have higher levels of circulating inflammatory cytokines including C-reactive protein, IL-8 and TNFα [12], which have been shown to relate to disease severity [13]. This up-regulation of cytokines also relates to low body mass index and peripheral muscle dysfunction [14]. These same inflammatory markers and cytokines can be found in patients with vascular disease and diabetes [15], and clustering of chronic inflammatory diseases is recognized in patients with COPD [14]. The presence of this systemic inflammatory syndrome and associated co-morbidities has a detrimental effect on morbidity and mortality [16].

In periodontitis, a complex interaction between inflammatory conditions has also been recognized. Again, a local inflammatory process is present in response to bacteria, but increased levels of systemic inflammation are also recognized, with higher circulating pro-inflammatory cytokines including C-reactive protein and TNFα [17]. Patients with severe chronic periodontitis have an increased risk of developing cardiovascular disease, thought, in part, to be due to the effect of the systemic cytokines, but also bacterial products, on vascular endothelial cells, resulting in the development and progression of atheroma and vascular plaque [18]. There is evidence that chronic periodontitis is also associated with an increased likelihood of stroke [19], osteoporosis [20], diabetes [21] and rheumatoid arthritis [22], through variations of the same mechanisms related to the general systemic inflammatory milieu.

It is unclear whether the relationship between these chronic diseases represents ‘overspill’ of local inflammation from one organ into the systemic circulation, or a systemic inflammatory process affecting multiple organ systems. This article reviews the available epidemiological and pathophysiological evidence to date and will determine whether a basis for an association exists between COPD and periodontitis, and, if so, the implications for further investigation and treatment.

A PubMed search was performed using the terms ‘COPD’, ‘emphysema’ and ‘periodontitis’, as well as ‘epidemiology’ and ‘neutrophil’. Publications were generally confined to the last 10 years, but older significant publications were not excluded. Relevant articles identified from the reference lists of articles identified by the initial search strategy were also included.

## Discussion

### Epidemiology of COPD and periodontitis

In addition to the similarities of pathological tissue destruction alluded to earlier, both periodontitis and COPD share similar risk factor profiles. Smoking is a well-known significant risk factor in COPD, with around 80% of patients with the disease being current or previous smokers [23]. COPD is also associated with age, with lung function declining from early adulthood [24]. Typically, there is also an association with male sex, although previously this mainly reflected smoking and working habits. However in recent years, the incidence has risen in females, reflecting increased smoking habits leading to a more even sex distribution of the disease. There is even some evidence that females may have a greater pre-disposition to COPD [25]. Although no bacteria or other microorganisms themselves have been linked to the development of COPD, lung infections are thought to be important in the progression of the disease, either by their chronic effects on airway inflammation [26] or acute effects on the rate of lung function decline following exacerbations [27].

Smoking has also repeatedly been shown to be a significant risk factor for the development of periodontitis [28,29]. This response is dose-dependent, with ‘heavy’ smokers twice as likely to lose connective tissue compared to ‘light’ smokers [30]. As with COPD, susceptibility is also greater in males and the elderly [31]. Additional risk factors include diabetes, especially in patients with type 1 disease and with increasing duration of a diabetes diagnosis [32]. Finally, other risk factors such as low socioeconomic status have been found to relate both to COPD outcomes [33] and the development of severe periodontitis [34], and may also account for some differences in disease presentation.

The hypothesis that there may be a pathophysiological link between these two chronic diseases was first proposed in the 1990s [10]. In the years that followed, retrospective data were analyzed investigating a possible relationship between dental health and respiratory disease. In the United States, information collected by the National Center for Health Statistics from over 20,000 individuals randomly sampled in the 1970s was analyzed for such associations. Amongst this sample, Scannapieco *et al*. identified 386 individuals who had reported an acute or chronic respiratory condition and had also undergone a dental examination [35]. An association was also noted linking patients with chronic respiratory disease to worse oral health. After performing logistic regression and adjusting for variables including smoking, it was found that those in the worst quartile for oral health had a 4.5 estimated odds ratio for having a physician-confirmed chronic respiratory condition. Subsequently, the same research group repeated the cross-sectional, retrospective study design using a different health survey of 13,792 patients [36]. In this second study, COPD was defined by responses to a questionnaire rather than by objective spirometric measurements, although these were taken. The authors found that lung function was lower in those individuals with poor periodontal health, and the most severe periodontitis scores conferred an odds ratio of 1.45 of reporting concurrent COPD.

Using a more robust study design, Hayes *et al*. looked longitudinally at a dataset of army veterans spanning 25 years [37]. A spirometric definition of a forced expiratory volume in one second (FEV_1_) of less than 65% predicted was used for COPD, and radiographic alveolar bone loss was used as a marker of periodontitis. After adjusting for co-variates, they found that for each 20% increment in alveolar bone loss from baseline, there was a 60% increase in the risk of COPD. It is worth noting that this definition of COPD is not diagnostic, as a reduced FEV_1_ is a feature of multiple obstructive and restrictive respiratory diseases. Assessment of symptoms and wider consideration of spirometric measures are required to diagnose COPD [38].

Similar associations have been identified in subsequent studies [39-41], some using more robust and internationally recognized definitions of disease and measuring multiple biometric indices [42]. A relationship between worse periodontal disease and increased severity of COPD has also been suggested [43]. These studies have predominantly come from China in recent years, where both a possible association between the prevalence of the two diseases and an assessment of health behaviors and quality of life have been assessed at the same time to gain a more complete understanding of the relationship [44]. For instance, in a case–control study of around 600 patients with and without COPD, the authors proposed that the relationship between COPD and periodontitis reflected oral health behaviors [45]. The authors confirmed that patients with COPD had worse markers of oral health including higher plaque index and worse periodontal attachment levels, but also had worse oral health behaviors such as tooth brushing frequency, use of dental floss and number of dental visits. This raised some concern about the interpretation of previous studies where such confounders were not evaluated in multivariate analysis. It may be the case that oral health behavior is a surrogate for poor general health behavior. A further study looked at the relationships between pathological and behavioral traits in COPD exacerbations and found a significant positive relationship with both poor oral health behavior and poor hygiene behavior [46]. This raises the possibility that oral health and health behaviors themselves may be central to the ‘frequent exacerbator’ phenotype of COPD, and hence suggests behavioral strategies that may impact the clinical and financial burden of such episodes.

Recent studies have adopted a different approach by investigating shared pathological features. The protease/anti-protease hypothesis, in which emphysema is caused by an imbalance of destructive proteases and their inhibitors (see heading ‘The protease/anti-protease imbalance’), has been widely accepted as a component of the pathophysiology of COPD. Periodontitis has also been studied with this hypothesis in mind [47]. Levels of matrix metalloproteinase (MMP) in saliva and serum of patients with mild COPD showed that serum neutrophil-specific MMP-8 (implicated in periodontal disease) was raised, although there was no statistically significant difference in saliva samples compared to patients without COPD. Though interpreted as a negative study, systemic inflammatory mediators described more recently in samples obtained from the crevicular cleft [48] were similar to findings in the lung in COPD [49].

The nature of the association between periodontal disease and COPD remains uncertain, especially as COPD is a generic term and has often been classified as present in periodontal studies without the required confirmatory spirometric measurements (as per guidelines from the National Institute of Clinical Excellence) [38] or pathological studies. There is also inconsistent assessment of periodontal health, utilizing disparate diagnostic criteria, and some element of selective analysis is required to identify associations. A recent review article noted the evidence for an association between COPD and periodontitis as ‘poor’ and showing, at best, only a weak association [50]. Other studies have also found mixed results with no significant differences in periodontal variables between COPD and non-COPD groups, except for probing depth that was greater in the non-COPD group [47]. In addition, whilst some measures of periodontal status may be significantly worse in COPD, other perhaps more relevant measures such as clinical attachment level have been unaffected [45]. It is clear that controlling for confounders is essential, but may be difficult especially if they relate to health behavior. There remains concern that, despite using multivariate analysis, residual unaccounted issues such as smoking may still exist in the epidemiological studies [51,52]. Moreover, the majority of studies are cross-sectional and although the authors speculate on causality, such observations permit little conclusion other than an association that is more common than expected for each disease alone. However, whilst some meta-analyses speculate that periodontal disease significantly increases the risk of COPD via mechanisms independent of the usual, recognized risk factors [53], this remains an area in need of high quality research.

### Pathophysiology in COPD and periodontitis

Although this review aims to discuss neutrophil phenotype and behavior as a shared final pathway in the pathological process, it would be naïve to neglect the roles of other cells in the pathophysiology of the diseases. The details of all the complex mechanisms in these diseases, however, are beyond the scope of this article, but many excellent reviews that cover these aspects are referenced below. Macrophages are another key inflammatory cell of the innate immune system that produce cysteine proteinases and MMPs that can degrade the supporting matrix of lung parenchyma, potentially causing emphysema [54]. They can also secrete increased levels of IL-4 and IL-13, worsening mucus production and exacerbating airway hyper-reactivity [55]. Dendritic cells form a link between the innate and adaptive immune responses, presenting antigens to activate immune cells in both conditions [56,57]. Auto-immunity is a potential mechanism of disease development and has been demonstrated in both COPD [58] and periodontitis [59], considered to be due in part to the production of antibodies to citrullinated proteins, where smoking is thought to play a major etiological role [60]. However, as the most abundant leukocyte with the greatest proteolytic capability, the neutrophil is thought to be the most important cell in the pathogenesis of both diseases [8,61], and it is the function and variations in phenotype of this inflammatory cell that this article will explore further.

### The neutrophil at the center of a shared pathological pathway

Neutrophils were amongst the first immune cells to be identified in the late 19th century, and are the most abundant of the white blood cells. They are also amongst the shortest lived, circulating for only a few hours [62]. They play a key role in the innate immune system, detecting and defending against potentially harmful organisms.

As a key effector cell in inflammation, neutrophils are found in increased numbers in COPD and periodontitis. In COPD, the hypothesis of a central role for the neutrophil has been based on extensive, though to date indirect, observations. Abnormal neutrophil functions are a feature of patients with COPD that may influence protease-/reactive oxygen species (ROS)-mediated damage. The neutrophils isolated from patients with COPD have a greater destructive potential [63], greater spontaneous adhesion to epithelial cells under flow [64], and different and more tortuous migratory dynamics than those from appropriate controls [65], which may account for their destructive potential. Bronchial tissue from patients with COPD contains increased numbers of neutrophils [66] as do the airway secretions of patients with chronic bronchitis [67]. However, fluorodeoxyglucose positron emission tomography scanning has shown that the activated neutrophils co-localize with areas of emphysema [68] and the amount of neutrophil elastase present in lung tissues correlates with the severity of emphysema [69], suggesting that this is a central process at least in the emphysema COPD phenotype.

This information is currently lacking in periodontitis although neutrophils also predominate as the inflammatory cell in gingivitis [70]. Their function is being increasingly investigated as a pathophysiological mechanism in chronic periodontitis [71] as well as COPD [65,72]. Whether the neutrophils have the same characteristics in periodontitis as in COPD, and thereby a common pathophysiological pathway, has yet to be studied. However, as part of their recruitment to lung or gingival cleft, activated neutrophils release a variety of mediators including proteases and oxygen radicals, which have been implicated in the tissue damage and inflammation characteristic of both diseases.

### The protease/anti-protease imbalance

As indicated above there is much data to support the protease/anti-protease imbalance pathway as a key process leading to tissue destruction in COPD. The basis of this hypothesis arose in the 1960s, when patients with deficiency of α_1_-antitrypsin (AAT) were noted to be specifically susceptible to the development of early onset emphysema disproportionate to smoking history [73]. AAT was subsequently shown to inhibit neutrophil elastase, an enzyme present in high concentrations in neutrophil azurophilic granules and a potent degrader of elastin, collagen and laminin. In the 1970s, this protease was found to induce emphysema when instilled into the lungs of animals [74]. In support of this as a mechanism, broncho-alveolar lavage fluid from patients with COPD showed a relationship between the elastase and anti-elastase imbalance and the extent of emphysema [75]. Surgically resected emphysematous lung tissue showed immunolocalization of neutrophil elastase in the lung interstitium in association with interstitial fibers, and displayed a quantitative relationship to pathological severity [69]. Subsequent work has shown that other proteolytic enzymes contained in neutrophil granules such as proteinase 3 and cathepsin G also have the potential to induce many of the pathological changes observed in COPD, not just emphysema [76].

**Figure 2:**
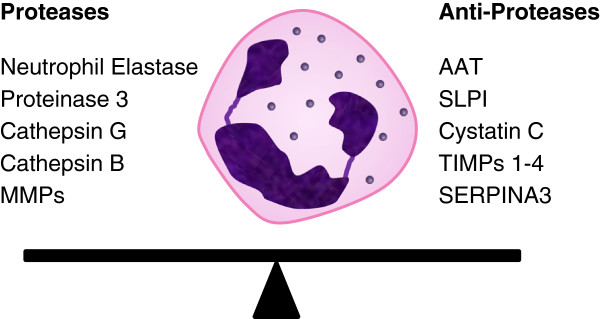
**The role of proteases and anti-proteases in tissue damage.** A complex balance exists between proteases and anti-proteases that determines the presence and extent of connective tissue damage. The interplay is made more complex by interactions between various molecules. As well as being inactivated by AAT, neutrophil elastase is also inhibited by SERPINA3 and SLPI. However, neutrophil elastase has the ability to inhibit TIMP1-4 (inhibitors of MMPs) and MMPs can inactivate AAT. This complex interaction of activation/inactivation means that interpreting the balance of proteases and anti-proteases is far from straight forward. AAT, α_1_-antitrypsin; MMPs, matrix metalloproteinases; SERPINA3, serine protease inhibitor gene; SLPI, secretory leucocyte protease inhibitor; TIMP, tissue inhibitors of metalloproteinase.

In addition to these neutrophil serine proteases listed above, there is increasing recognition of the role of MMPs in COPD. These enzymes are also released from neutrophils and other inflammatory cells such as macrophages. Several have been found in a higher concentration in the emphysematous lung [49], and knockout and transgenic mice for these MMPs suggest that they play a role in emphysema [77]. These enzymes are also controlled in health by specific tissue inhibitors of metalloproteinases, of which there are four subtypes [78], adding further complexity to the activation/inactivation of proteolytic elements. It is likely, however, that the neutrophilic serine proteinases, MMPs and cysteine proteinases interact in a proteolytic and inflammatory cascade, which needs careful characterization to identify the key drivers of the tissue destruction [79]. Some of the components of this cascade are summarized in Figure 2.

Though a protease/anti-protease balance is less well studied in periodontal disease, it makes sense to consider this process in a disease where loss of collagen in connective tissue is pathognomic and there is a body of evidence to support it as a key pathological process. Early studies found up to seven times the concentration of neutrophil collagenase and elastase in the crevicular fluid of patients with chronically inflamed gingivae [80]. This discovery ignited an interest in whether there may be a shared link with emphysema and, as in the latter disease, investigators started to study the main serine proteinase inhibitor, AAT [81]. Although both neutrophil elastase and AAT were present in increased amounts in patients with periodontitis [82], an imbalance in favor of the enzyme was also implicated in the disease process. It was logical to assume that such a process (as in COPD) would be amplified in patients with AAT deficiency. Indeed, an early study found that deep periodontal pockets were more prevalent in an AAT-deficient group compared to matched, healthy controls, though there was no difference in attachment loss [83]. However, a more recent study failed to find any difference in periodontal parameters in an AAT-deficient group compared to controls [84]. Nevertheless, this concept was further explored, with the theory that despite an increase of local AAT it could also be inactivated in periodontitis [85], leading to a functional imbalance and hence continued enzyme activity. Assays subsequently showed that inactivation of AAT was significantly greater in the crevicular fluid of patient with periodontitis although the mechanism underlying this was unknown [86]. In addition, the complexity of interactions with other enzymes and inhibitors, as in COPD, has also been proposed as relevant in periodontitis [87].

Although more work is needed in this area, the idea that protease/anti-protease imbalance is a cornerstone process in the connective tissue destruction seen in emphysema and periodontitis seems intuitive. No support for this has been found by studies of AAT deficiency and periodontitis, but it is likely that the sample sizes were too small and the question addressed too superficially to provide a definitive answer.

### Oxidative stress in COPD and periodontitis

The lung has a surface area of approximately 70 square meters, and as a result has a huge exposure to sources of free radicals and ROS such as in tobacco smoke and air pollution [88]. Cigarette smoke contains large amounts of oxidants and free radicals that are a major part of the oxidative stress endured by host cells [89]. In addition to these exogenous sources, activated inflammatory cells such as macrophages and neutrophils generate ROS in response to environmental insults, as well as in response to bacteria and their products [90]. This process has received much attention in COPD and is also thought to be central to the pathological processes in periodontitis, where again the activated neutrophil has been implicated as the source of oxidative stress [71].

**Figure 3:**
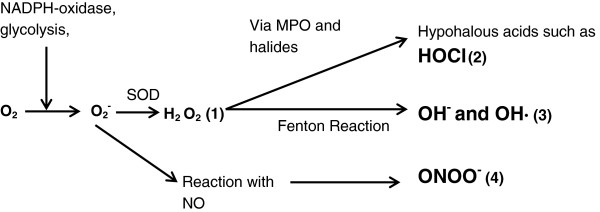
**The formation of reactive oxygen species within inflammatory cells.** Activation of the membrane bound NADPH-oxidase causes the reduction of the oxygen molecule to the superoxide anion, which dismutates, either spontaneously or via superoxide dismutase, to form hydrogen peroxide (1). Reactions with halide elements such as chlorine generate potent oxidants, for example hypochlorous acid (2). Alternatively, conversion to hydroxyl anion/radical can occur, catalyzed by iron in the Fenton reaction (3). Superoxide can also combine with nitric oxide to create the potent oxidant peroxynitrite (4). H_2_O_2_, hydrogen peroxide; HOCl, hypochlorous acid; MPO, myeloperoxidase; NADPH, nicotinamide adenine dinucleotide phosphate-oxidase; NO, nitric oxide; O2, oxygen; O2^-^, superoxide; OH^-^, hydroxyl anion; OH·, hydroxyl radical; ONOO^-^, peroxynitrite; SOD, superoxide dismutase.

ROS formation happens inside every human cell as a bi-product of normal metabolism. Oxygen is used in glycolysis to form pyruvate, and along the enzyme cascade an electron leak reduces oxygen to the superoxide anion O_2_^-^, which is the first step in the formation of further ROS. In inflammatory cells, such as neutrophils, ROS can also be generated as part of the respiratory burst. As nicotinamide adenine dinucleotide phosphate-oxidase assembles on the plasma membrane, it reduces molecular oxygen to superoxide, which either spontaneously forms hydrogen peroxide, or is more rapidly catalyzed to hydrogen peroxide by superoxide dismutase. Myeloperoxidase catalyzes the oxidation of halides, creating potent hypohalous acids such as hypochlorous acid. The Fenton reaction oxidizes ferrous iron to its ferric state, causing the reduction of hydrogen peroxide and creating a hydroxide radical and hydroxide anion (both potent oxidants). In addition to these products, reactive nitrogen species are also formed in inflammatory cells [91], utilizing nitric oxide formed by nitric oxide synthases. Nitric oxide has important physiological roles, such as vasodilation. It is lipophilic and enters cells, interacting with superoxide to produce other potent oxidants such as peroxynitrite [92]. Figure 3 illustrates the processes that form these physiologically, as well as pathologically important oxidant species.

As well as playing a role in bacterial killing, ROS can damage host tissues, making them more susceptible to proteolytic degradation. They can also amplify the inflammatory process, including lipid peroxidation [93], leading to breakdown of lipids in the cell membrane and direct DNA damage [94,95]. Superoxide induces the generation of inflammatory cytokines, including TNF-α [95], via redox-sensitive transcription factors such as nuclear factor kappa-light-chain-enhancer of activated B cells. Moreover, superoxide can increase chemoattractant production [96] and hydrogen peroxide can up-regulate the expression of adhesion molecules [97], increasing inflammatory cell infiltration. In addition, peroxynitrite can, potentially, augment protease damage to host cells by inactivating inhibitors such as AAT [98].

To prevent damage to host tissues by these potent molecules, the action of oxidants is counteracted by antioxidants, such as catalase and glutathione peroxidase, which ‘break down’ hydrogen peroxide into water, oxygen and benign hydroxyl compounds. In general, when there is ROS excess, the resulting oxidative stress leads to tissue damage. Conversely, when antioxidant defenses predominate, the oxidant state facilitates tissue repair.

Oxidative stress is a feature of both COPD and periodontitis and thus implicated in the pathophysiology of both. In COPD, current smoking habit is associated with higher hydrogen peroxide levels in exhaled breath condensate than both ex-smokers with COPD and non-smokers [99]. Statistically significant increases of 8-hydroxyl-2’-deoxyguanosine, which is a marker of oxidant-induced DNA damage, were seen in patients with COPD compared with smokers without COPD and healthy controls [100]. In addition, the products of lipid peroxidation are higher in patients with COPD [101] and COPD is associated with polymorphisms of the superoxide dismutase 3 gene, and hence its function [102]. There is also a positive relationship between sputum neutrophils and hydrogen peroxide levels in patients with severe COPD, suggesting these cells are a major source of the oxidant [103]. Finally, COPD neutrophils have greater oxidant production than those of smokers with normal lung function and non-smoking control individuals [104]. All these observations suggest that oxidant stress could be a central feature of COPD.

There is also evidence of hyperactive and reactive neutrophils in periodontitis. In localized aggressive periodontitis, peripheral neutrophils produce increased amounts of ROS after exposure to chemoattractants [105]. Indeed, in chronic periodontitis, even unstimulated neutrophils had greater spontaneous ROS production, as detected by chemiluminescence, than cells from control individuals [106]. In addition, greater amounts of ROS were produced in patient cells following exogenous stimulation by a known periodontal pathogen, *Fusobacterium nucleatum*, compared to cells of appropriate controls. Furthermore, the redox balance is probably disturbed by defects in local levels of antioxidants, as the gingival crevicular fluid contains less glutathione in patients with periodontitis than controls [107]. Neutrophil protease release following stimulation has yet to be studied in periodontitis, but has been shown to be increased both pre- and post-stimulation in emphysema [63].

### Neutrophil extracellular traps

Neutrophil extracellular traps (NETs) are an active form of cell death (NETosis), whereupon stimulated neutrophils release cellular contents including histones and decondensed chromatin. Once in the extracellular space, these components have the ability to trap microorganisms and expose them to high local concentrations of destructive enzymes [108]. They function as part of the innate immune system so that bacterial killing can continue even after the death of the neutrophils.

**Figure 4:**
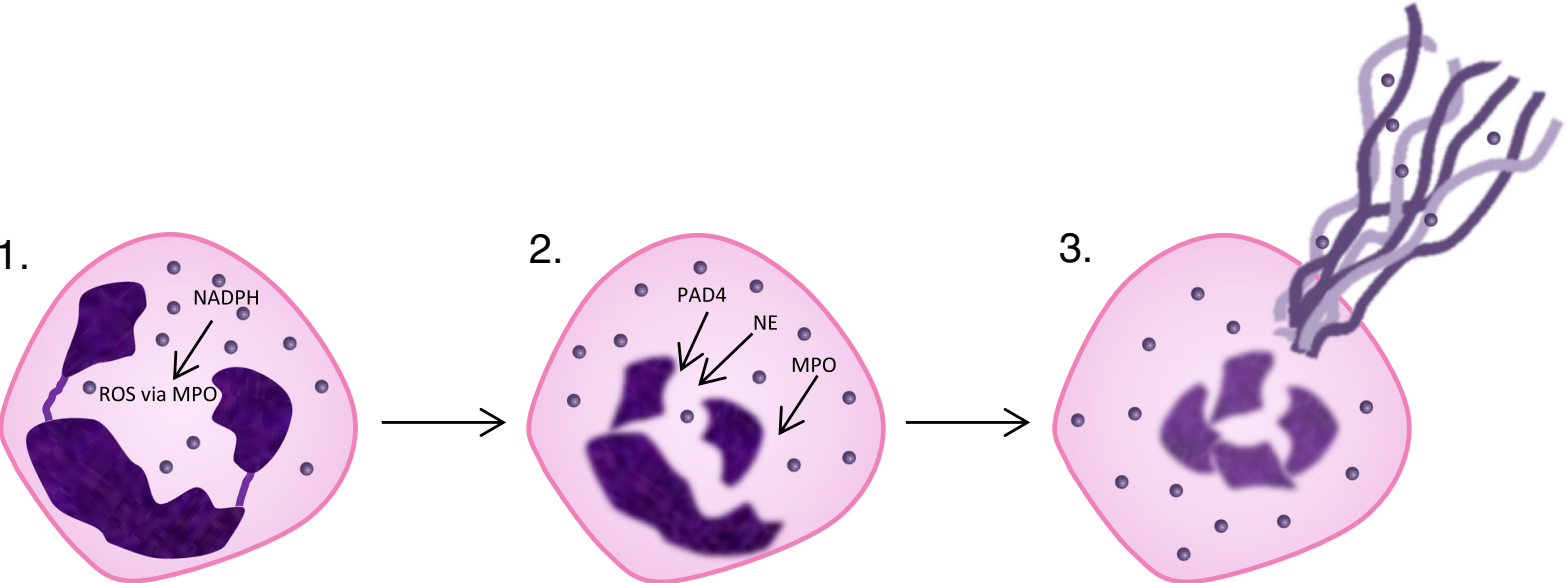
**Schematic of neutrophil extracellular trap release.** Activation of NADPH-oxidase causes reactive oxygen species generation (1). Granule components neutrophil elastase and myeloperoxidase are released. The enzyme peptidyl arginine deiminase, type IV is activated and acts to hypercitrullinate the nuclear chromatin (2). The nucleus disintegrates and DNA/histones are extruded through the cell membrane resulting in NETosis (3). NET components are able to trap microorganisms and expose them to increased local concentrations of destructive enzymes. NET, neutrophil extracellular traps.

ROS have been implicated in the formation of NETs [109], in particular myeloperoxidase, which catalyzes the conversion of hydrogen peroxide into hypochlorite. Deficiencies in myeloperoxidase, as well as absence of its substrate chloride, decreases NET formation and limits bacterial killing [110,111] and hypochlorous acid seems to be most important for NET formation after cell death [112]. A schematic of NET release is shown in Figure 4.

Extracellular DNase production is a characteristic of certain bacteria and bacteria present in patients with periodontitis, including *Porphyromonas gingivalis* and *F. nucleatum*, are a source of DNase and can degrade NETs [113], providing a potential bacterial advantage. It is hypothesized that the ability to neutralize the effectiveness of NETs augments microorganism virulence and may play an amplification role in the pathogenesis of chronic periodontitis.

NETs have also been implicated in host cell cytotoxicity [114] and as a possible mediator in lung inflammation [115]. Whether NETs and their degradation play a role in the pathogenesis of COPD is not known.

### Treatment effects

If the hypothesis of overspill of local inflammation into the systemic circulation leading to distant co-morbidity is correct, it is possible that effective treatment of one condition would have an appreciable effect on the other. So far, few trials have been conducted to test this hypothesis. It has already been mentioned that there is a positive relationship between oral health behaviors, such as tooth brushing time and supra-gingival scaling, and COPD exacerbation frequency [46]. Recently, a prospective controlled group trial of periodontal treatment in patients with both diseases was undertaken [116]. Forty patients with COPD from chest clinics, with concomitant moderate or severe chronic periodontitis, were selected. Half received initial periodontal therapy, whilst the others had no treatment. Over the subsequent 12 months, the treatment group had a decline in their median exacerbation frequency from three to two, whilst the control group had an increase from two to three. Though this is an interesting result, the groups were not randomly selected and the sample size was small. Nevertheless this early observation provides support for a distant effect of a local inflammatory process, and is worthy of further investigation with more carefully designed and appropriately powered studies, particularly given the circumstantial evidence outlined above.

## Summary

COPD (especially the emphysema sub-type) and periodontitis are believed to share a similar pathophysiology, namely inflammation and the destruction of the local connective tissue. There is evidence that the neutrophil is a key cell in the inflammatory response to both diseases, and that its proteases and ROS can propagate inflammation and cause damage to the components of the connective tissue. To cause the disease state, an imbalance has to occur between these degradative proteins and their inhibitors, and current research is focused on the factors that lead to this imbalance. Neutrophil function is clearly abnormal in COPD and may be central to the pathophysiology but it is not known whether a similar defect is present in patients with periodontitis. Further studies looking at the existence of shared pathological processes and the effect of specific interventions in either disease on the other will shed more light on any link between the conditions. However, this will require appropriately powered randomized controlled trials to investigate causality and understand the pathological basis of the diseases.

## Abbreviations

AAT: α_1_-antitrypsin; COPD: Chronic obstructive pulmonary disease; IL: Interleukin; MMP: Matrix metalloproteinase; NETs: Neutrophil extracellular traps; ROS: Reactive oxygen species; TNF-α: Tumor necrosis factor α.

## Competing interests

The authors declare that they have no competing interests.

## Authors’ contributions

AU and RS conceived the manuscript outline. AU wrote the manuscript, which was critically reviewed by RS. Both authors read and approved the final manuscript.

## Authors’ information

AU is a clinical research fellow in the University of Birmingham and at the Queen Elizabeth Hospital, Birmingham; RS is Professor of Medicine at the University of Birmingham and at the Queen Elizabeth Hospital, Birmingham.
